# Lectures on the Exploration and Treatment of Diseases of the Chest

**Published:** 1840-06-20

**Authors:** W. W. Gerhard


### MEDICAL EXAMINER.

DEVOTED TO MEDICINE, SURGERY, AND THE COLLATERAL SCIENCES.

No. 25.] PHILADELPHIA, SATURDAY, JUNE 20, 1840. [Vol. III.

### LECTURES ON THE EXPLORATION AND TREATMENT OF DISEASES OF THE CHEST.

BY W. W. GERHARD, M. D.

Lecture VI.—*Cough—Expectoration.*

Cough is produced in diseases of the thorax from two causes—the accumulation of liquid in the bronchial tubes, and the sympathetic irritation caused in the larynx by pain or stricture in the chest. In the former variety, the cough is useful, and is productive of relief to the patient, in the latter it is often a cause of aggravation of symptoms. The true excretory cough occurs only in the diseases of the bronchial mucous membrane, and of the parenchyma of the lungs which directly communicate with this membrane. The irritative cough takes place not only in the earlier stages of inflammation of the bronchial tubes, and of disorders of the parenchyma and serous membranes which do not communicate with the bronchi, but it is also a frequent dependent upon diseases of the heart, and even of the stomach, and in many cases is caused by a disordered condition of the nervous system, which is totally foreign to the chest. You perceive, therefore, that the causes of the irritative cough are extremely various, and that the cough itself, in many cases, throws but little light upon them.

I shall now attempt to define to you the varieties of cough and of the expectoration, which are closely connected together.

*The dry or irritative cough.*—The term irritative may properly enough be applied to this variety, which is nothing but a short and quick cough,—that is, a short and rapid expiration, which is the essential character of cough. The term dry cough is so well known as the designation of this variety, that it is universally understood. It is followed by no real secretion; there is sometimes an expectoration of the small quantity of mucus, which is naturally found in the fauces and bronchi. The diseases of the lungs in which it occurs are the early stages of phthisis, and certain cases of serous inflammation. It is also an attendant upon the elongation and inflammation of the uvula, and may cease abruptly after its removal. In diseases of the stomach and bowels, and in affections of the mucous membranes of the abdomen as well as in peritonitis, the same variety of cough is observed. Indeed, you may generalize the subject much farther, and say that the short, dry cough, is the most frequent form of irritative cough, and the most persistent; and that, although in itself it is of no moment, it is often the sign of a commencing disease of the thorax. On the other hand, your knowledge of the circumstances which give rise to a dry cough, must lead you to look for other causes of it than the diseases of the chest,—and after your physical examination has taught you that there is no important lesion in the thorax, your next object will be to examine other portions of the body, and ascertain whether some disease of the abdominal viscera, or a mere nervous irritability, will not account for this cough.

There is another variety of cough which is not very unlike the dry; that is, the sonorous cough: this is always loud, and at times very ringing and clear, so as to be heard at a considerable distance from the patient. This variety belongs to many morbid conditions: it is found in the chronic dry catarrh, but chiefly in the earlier stages of ordinary acute catarrh, before secretion has commenced. In its most marked degree, however, the sonorous cough is not indicative of diseases of the lungs, but of many and various conditions of this morbid nervous action; and, as you may readily suppose, it is most apt to occur in young girls, who are much more subject than any other class of individuals to diseases attended with deranged nervous action. Hence the cough is very irregular in its indication; and although when it is of recent occurrence and short duration, it is nearly always connected with disorder of the bronchial tubes,—yet, when chronic, it is most frequently either a true nervous cough, or an attendant upon chronic diseases of the larynx, especially those in which there is a morbid growth which projects into the rima glottidis, and acts as a constant cause of irritation. This cough is therefore rather a matter which must exercise your sagacity, than a correct indication of any special disease.

The *suppressed cough* is, like the dry, a short cough; but it is checked by a voluntary effort of the patient; for as the act of coughing is, to a certain extent, independent of the will, a patient may arrest the violent expiration if he be aware that it will cause him much pain; hence the cough becomes suppressed in serous inflammations of the chest, where there is little or no secretion from the bronchi, and the pain is much more considerable than in ordinary cases of disease. In pertussis, the fear of exciting a violent fit of coughing will frequently cause it to be suppressed. In the early stages of pneumonia there is very little secretion into the bronchi; hence the necessity for cough and expectoration is but slight, while the accompanying pleuritic inflammation acts as in cases of simple pleurisy, and suppresses the cough.

The *laryngeal cough* is various in its character; still, as it depends upon thickening or ulceration of the larynx, the tone of the cough is stridulous and somewhat stifled; at times, almost whistling. In the advanced ulceration of the larynx, which constitutes laryngeal phthisis, the cough is alternately loud and whistling, and again almost aphonic. This variety of the cough is attended with a peculiar alteration of the voice.

The *loose*, or *mucous cough*, is well known as the cough which attends the resolution of acute bronchitis, and is therefore of favourable prognosis in this disease; it is connected with a free secretion into the bronchial tubes, and is of course accompanied by mucous rhonchus, and generally by expectoration. As there are many diseases in which there is an abundant liquid secretion into the bronchial tubes, the mucous cough is very far from being confined to bronchitis; it occurs also in the advanced stages of phthisis, in the third stage of pneumonia, hæmoptysis, &c. Hence, like most of the varieties of cough, it becomes useful as a sign, chiefly when combined with other symptoms.

In certain cases of large cavities from phthisis or gangrene, the cough sometimes is not merely mucous, but it is loud and rattling; that is, as it is caused by the free agitation of the air in a large cavity, it partakes of the characters of the cavernous respiration, and differs in being much louder and more gurgling from the ordinary mucous cough.

The *spasmodic cough* is the last variety of cough which is sufficiently characterized to admit of a separate description. The type of this variety is found in pertussis, in which disease the cough is more decidedly spasmodic than in any other. But there are numerous other cases of disease, especially lesions situated about the larynx, which are attended with a severe cough, returning in paroxysms, and sometimes accompanied with a noisy, whooping inspiration. Although it is most frequent in obstructions about the larynx and upper part of the trachea, the enlargement of the bronchial glands will often give rise to it, and the peculiar cough is sometimes a valuable diagnostic sign in an affection which is always obscure. In certain cases of asthma the cough recurs in paroxysms which are often attended with a noisy inspiration. In general terms, you may state, cough does not bear an accurate relation to the extent of the pulmonary lesion; frequently the cough seems to be almost in inverse proportion to the mass of parenchyma involved in the disease. For if a large portion of the lungs be rendered unfit for the performance of the respiration, the patient cannot make the forcible expiration necessary to produce a decided cough. It is rather a sign of laryngeal and tracheal irritation, than of deep-seated pulmonary disorder. The cough is of less value as a sign in the aged than in those enfeebled by disease, or than in other patients, for in them it may be wanting throughout the whole course of a grave disease: the same remark is applicable to young children, who cough much less frequently than those who are older. In diseases of the lungs in general, the cough may completely cease if the brain becomes seriously involved; for a cerebral disorder renders a patient unconscious of the irritation, which, under ordinary circumstances, would give rise to severe cough. Secondary inflammation of other organs, as the stomach and bowels, sometimes produce a similar effect, but to a much less degree: this is in accordance with the general pathological law, that a severe intercurrent inflammation will obscure, and, to some extent, replace the symptoms of the primitive affection.

### THE EXPECTORATION.

The expectoration is less frequent in diseases of the chest than the cough; but its signs are more definite, and in some cases they afford very accurate indications of pulmonary disease. As a general rule, the sputa come from the lining membrane of the bronchial tubes, and form cavities or softened portions of the parenchyma, which communicate directly with the bronchi. Hence, their value as positive signs is chiefly confined to the diseases which affect the mucous membranes of the chest. The sputa, however, may contain other liquids besides the ordinary secretions of the mucous membranes, such as blood, tuberculous and calcareous matters.

The secretion of liquids in the bronchi is necessarily independent of the will, but the expectoration is a voluntary act. It is performed imperfectly when a person is averse to making the necessary muscular exertion, on account of the pain it may give him, or other reasons; there are no sputa when the feebleness of the patient prevents his making an effort. For similar reasons, children below the age of six years do not expectorate; they do so but rarely until the age of puberty. In very old people the expectoration is rare, and not proportioned to the extent of the disease.

When the sputa are not copious, they are chiefly expectorated in the morning, on waking from sleep, during which they accumulate in the bronchi. When the sputa are copious, but the expectoration causes pain, they are also retained in the lungs until a paroxysm of coughing comes on, and they are discharged in large quantities.

Except in the cases above mentioned, the sputa are rarely wanting during the whole course of a disease, but they do not usually assume their characteristic appearance until the disease is sufficiently advanced to be recognized by the more certain physical signs. In some exceptional cases the sputa are pathognomonic, when the physical signs are doubtful, on account of the remote situation of the lesion or the state of the surrounding tissue.

1. *Of the quantity of the expectoration.*—It is small when it does not exceed a wine-glassful in the twenty-four hours; moderate, when from two to six fluid ounces; large, from six ounces to a pint, and very large if more than a pint. In descriptions of the sputa, it is advisable to state the quantity.

2. *Of the colour.*—The saliva and the mucus of the bronchial tubes are transparent; and may be more abundant than usual. A higher, or rather more prolonged degree of inflammation of the bronchial mucous membrane, gives a whitish colour to the sputa, if the catarrh pass into resolution; or, if it assume a chronic form, the sputa are yellowish, and frequently of a greenish tinge, and altogether opaque. In acute inflammations of the air-vescicles and of the minute bronchial tubes, the sputa are at first transparent and colourless, but soon become tinged of an orange hue, or they are rust-coloured or of a bright scarlet colour. In inflammations of the lungs, with great prostration, the sputa are brownish, of a mahogany colour, or like that of stewed prunes.

3. *Consistence, and chemical composition.*—In general, the sputa, if colourless, are thin and very liquid; those that are yellow and opaque, are thick, and flow less easily. The shining transparent sputa of pneumonia are more viscid than any other, are often heaped up in the centre of the cup, and adhere strongly to its sides. In one variety of chronic catarrh, and in some affections of the tonsils, the matter expectorated is very small in quantity, and almost solid. The sputa frequently consist of two parts, one more solid, and the other nearly of the consistence of water. If much air be mingled with the sputa, they are light and frothy. The chemical nature of ordinary bronchitic sputa scarcely differs from that of the healthy mucus of the bronchial tubes, but if the inflammation be more advanced, the sputa are more opaque, and become more albuminous. The increasing thickness of the sputa is a sign of a tendency to resolution in acute bronchitis, which is but slightly influenced by the mucous expectoration of its earlier stages. When pus is mixed with the mucus, the consistence is immediately increased; the thick pasty sputa which occur in advanced stages of phthisis, in which the softening is very rapid, are very consistent, but adhere together less intimately than the sputa of pneumonia.

4. *Form.*—When the sputa are composed of simple mucus from the bronchial tubes, they run together, and form a mass which is perfectly homogeneous,—and when they become albuminous, they offer no peculiar form, but are generally composed of two parts—one consisting of the whitish opaque mucus, which, in the form of shreds, is diffused through the mass of the liquid, and the other more transparent. In some cases of bronchitis, especially of the chronic varieties, in which the sputa are more albuminous than in any other, the matter is moulded into the form of the smaller bronchi, and is expectorated in little cylinders, which are diffused through the secretions of the larger tubes. The viscid, transparent sputa of pneumonia, blend together perfectly well, and form a mass which is with difficulty separated into smaller parts; and the sputa, both of the early and later stages of this disease, are so nearly similar to those of different stages of bronchitis, that they can scarcely be distinguished from them. The form assumed by the expectoration of phthisis is similar to that of bronchitis in its early stages: after softening has been completed, the sputa are moulded in the cavities, and form irregular, rounded masses, with loose cottony edges; these constitute the nummular sputa; when the softening is very rapid, the sputa run together, and lose their nummular form. The sputa, in gangrene of the lungs, retain no peculiar form, but vary according to the consistence of the matter in different cases of the disease.

5. *Odour.*—Transparent sputa are without decided odour; the thick, yellow liquid has generally a faint, nauseous smell, which is very marked in cases of phthisis. Gangrene of the lungs is distinguished by a peculiar fœtor, sometimes gangrenous, at other times resembling the smell of moist plaster. Occasionally, a variety of chronic catarrh and of tuberculous phthisis, in their advanced stages, are attended with fœtid expectoration.

*Of the foreign matters mingled with the secretions of the bronchial tubes.*—Pus is often intermixed with the mucus secreted in bronchitis, phthisis, and the latter stages of pneumonia, when the sputa are said to be mucopurulent. Sometimes a portion of the pus is uncombined, and sinks to the bottom of the mass. Blood may be intimately combined with the sputa, as it is in pneumonia, when it communicates a general rusty or reddish tinge to them; or it may be mixed in streaks with the mucus, and still retain its florid red color; or, lastly, it may be unmixed with the bronchial secretions, when it constitutes hæmoptysis. The tuberculous matter may sometimes, though rarely, be detected in the sputa under the form of minute yellowish opaque grains, not often exceeding the size of a pin's head; this appearance coincides with the softening of the tubercles. Calcareous matter is sometimes, though rarely, observed when the tubercles are dry and contain much of the salts of lime. Portions of gray or dark pulmonary tissue have also been expectorated, after separation from the adjacent tissue. In cases of jaundice or pneumonia, complicated with disease of the liver, the sputa are sometimes tinged with bile. I have seen the expectoration composed almost entirely of pure bile from a fistulous opening between the liver and the lungs, following a wound of these organs.

I have confined these remarks on the expectoration chiefly to the text of a short work on physical diagnosis which I published a few years since. They might be much extended; but as the subject is one to which I shall be obliged frequently to recur when speaking of individual diseases, I do not wish to annoy you with unnecessary repetitions. Still, it is essential for you to acquire some idea of the general characters of the expectoration. The best method of examining the sputa is to direct the patient to spit in a white or transparent vessel; a common tumbler will do well enough for this purpose, and then inspect them within a few minutes after they are discharged.

The chemical analysis of the sputa has thus far led to few or no practical results; for the characteristic distinctions between the various forms of mucus, albumen, and pus, are extremely slight. Indeed, it is not necessary for you to investigate, or rather to attempt to investigate, these slighter differences in the expectoration, which were at one time regarded as important. Amongst these, are the numerous tests between pus and mucus, which were sought in order to decide upon the distinctive characters of phthisis and catarrh; all these were found more or less fallacious: the best are the most simple,—that is, the yellow, purulent colour of the expectoration, when pus is mixed with the mucus, for it is rarely found in a separate state. This very admixture is one of the reasons which must make it impossible to discriminate in all cases, as to the mucous or purulent character of the expectoration. The whole subject is now placed in its proper light: the expectoration furnishes us with a most valuable secondary means of diagnosis, but one less important than many other methods of investigation that have now come into general use.

There is another class of symptoms which may be almost classed among the local signs of thoracic diseases,—that is, the mode in which the movement of the chest is performed during the act of respiration. In reference to this part of the course I shall content myself with quoting the observations contained in the work to which I have previously alluded. You will better understand their value by a reference to the numerous illustrations which I give you on this subject.

ON THE MOVEMENT OF THE THORAX.

In health, the act of inspiration is performed partly by the elevation of the shoulders and ribs, and partly by the depression of the diaphragm. The passage of the air through the nostrils does not cause them to dilate evidently. When the respiration becomes difficult, the different muscles, whose action concurs in respiration, act irregularly, and much more forcibly than in a state of health. When there is dyspnœa, without pain in any part of the thorax, all the muscles concerned in respiration act with increased energy. The nostrils dilate widely, the shoulders and ribs are forcibly elevated, and the diaphragm depressed. In acute diseases, the degree of the dyspnœa is nearly commensurate with the extent of the pulmonary affection. In chronic diseases, this is by no means the case. There are even some instances in which there is extreme dyspnœa, but no appreciable lesion of the lungs. When there is acute pain in the sides of the thorax, or at the diaphragm, from inflammation of the serous membranes, the parts of the chest nearest to the inflamed pleura move less than they do in a state of health. The motion becomes free as soon as the pain subsides. If effusion of liquid occur into the pleura or the pericardium, the motion of the ribs at the corresponding part is impeded by the mechanical distension, though there be no acute pain. When the liquid is absorbed, and false membranes unite the two surfaces of the pleura, the dilatation of the diseased side is always imperfect. The diminished motion of the side of the chest, most affected in phthisis, depends upon the adhesions produced by the frequent inflammations of the pleura.

The number of the inspirations is generally from twelve to sixteen in the minute; but when the lungs or the pleura are much inflamed, the inspirations may increase to thirty or forty; and, when the disease is extremely violent, the number may be as high as sixty or seventy. This extreme frequency is most remarkable when all the serous membranes of the chest are inflamed at the same time. In acute diseases, the frequency of the inspirations is at first nearly proportioned to the violence of the affections; when they have lasted a certain time, the patient seems to accommodate himself to a diminished supply of air, and breathes less frequently. The respiration of children affected with diseases of the chest is very frequent, especially when the lobular pneumonia has extended to a large portion of both lungs. When the extreme frequency of the respiration in acute diseases has ceased, the inspiration remains more hurried than usual; sometimes it is performed in as short a time as the expiration—after which a pause ensues. In health, the time required for the inspiration is twice as long as that of the expiration, both in children and adults.

